# Supplementary material for: Extracellular Vesicles Secreted by Pre-Hatching Bovine Embryos Produced In Vitro and In Vivo Alter the Expression of IFNtau-Stimulated Genes in Bovine Endometrial Cells
Source: Int J Mol Sci. 2023 Apr 18;24(8):7438. doi: 10.3390/ijms24087438 (PMC10138918; doi:10.3390/ijms24087438)
Supplement: Supplementary file 1 [file ijms-24-07438-s001.zip › ijms-2330032-supplementary.pdf]

Supplementary Material

Extracellular vesicles secreted by pre-hatching bovine embryos produced in vitro and in vivo alter the expression of IFNtau- stimulatedgenes in bovine endometrial cells

Aguilera Constanza[1], Velásquez Alejandra E[1], Gutierrez-Reinoso Miguel Angel[1], Wong Yat Sen[1], Melo-Báez Bárbara[1], Cabezas Joel[1], Caamaño Diego[1], Navarrete Felipe[1], Rojas Daniela[2],Castro Fidel Ovidio[1], Rodriguez-Álvarez Lleretny[1*].

*** Correspondence:**
Rodriguez-Álvarez Lleretny
llrodriguez@udec.cl

1 Supplementary Figures

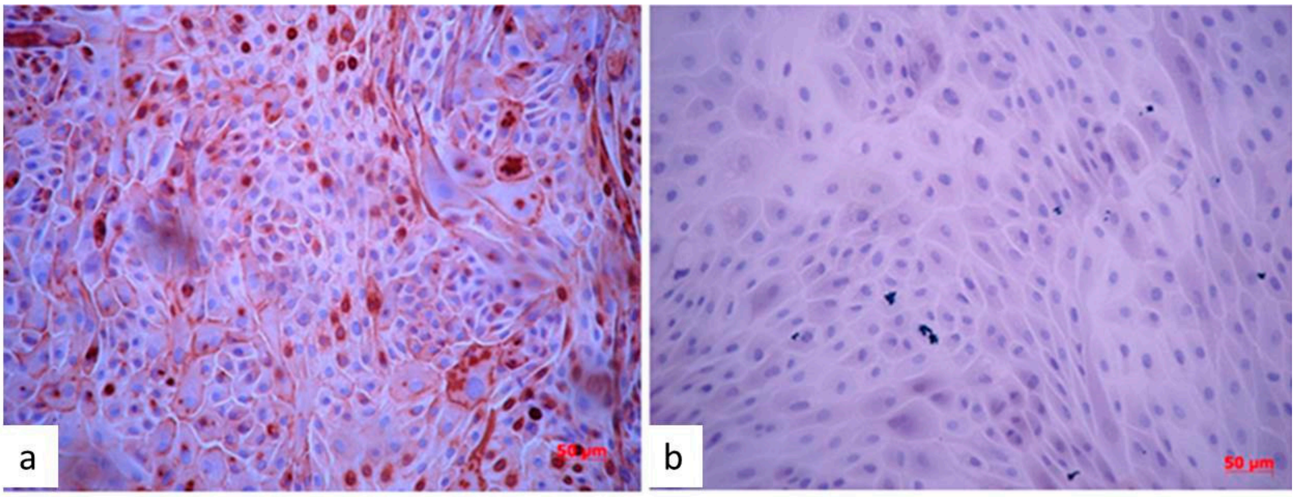

Figure 1S. Immunocytochemistry of cytokeratin. a) epithelial cells positive to cytokeratin , b) negative cells to vimentin stromal cell marker.

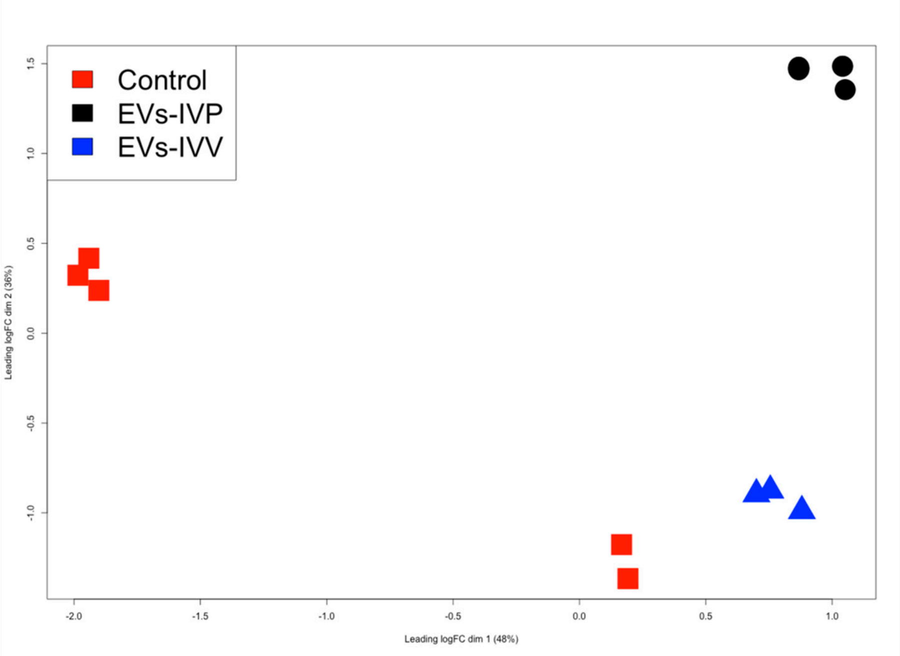

Figure 2S. Principal component analysis (PCA) of gene expression level in endometrial cells exposed to in vitro (EVs-IVP) or In vivo (EVs-IVV) EVs from bovine embryos. The PCA shows clusters of expressed genes that differ across the samples.

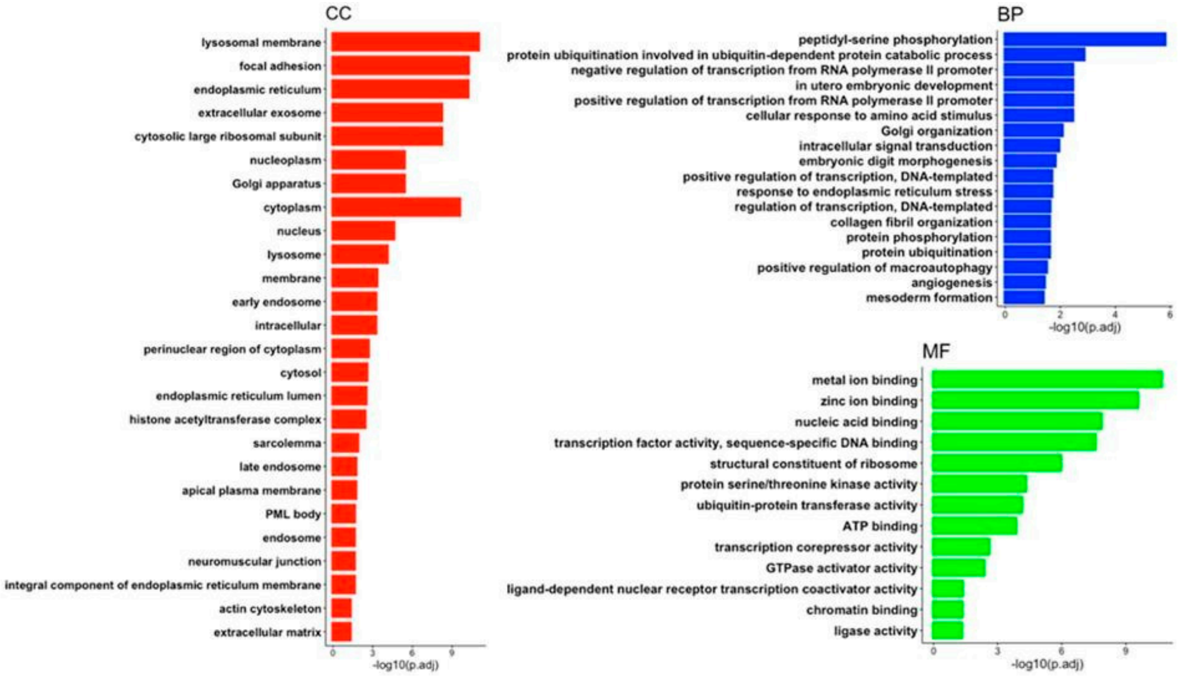

Figure 3S. G.O analysis based on DEGs between EVs-IVV and control. The significantly pathwayswere classified as biological process (BP), cellular component (CC) and molecular function (MF).

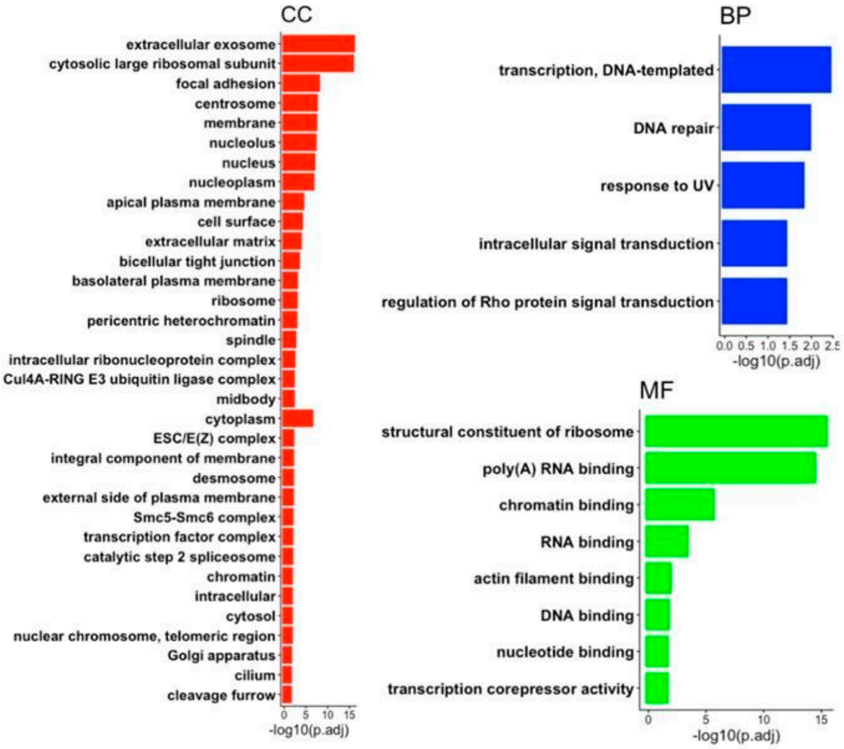

Figure 4S. G.O analysis based on DEGs between EVs-IVP and control. The significantly pathwayswere classified as biological process (BP), cellular component (CC) and molecular function (MF).

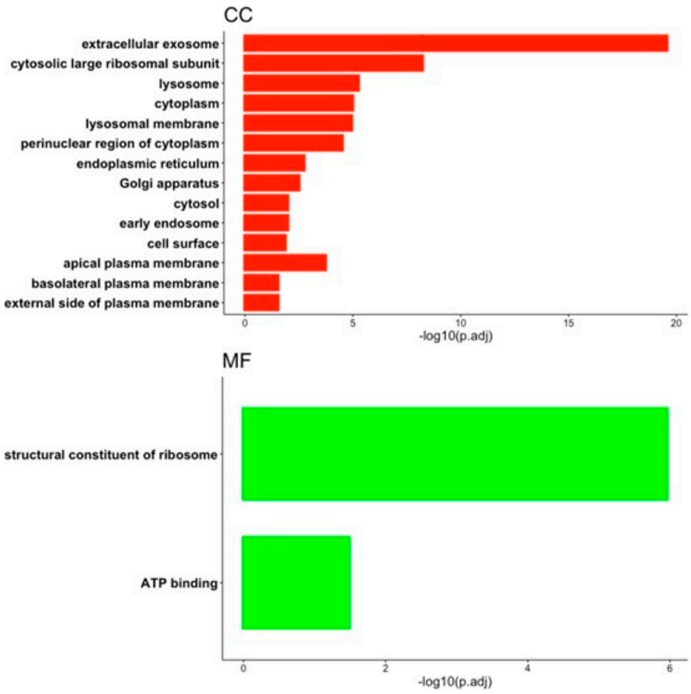

Figure 5S. G.O analysis based on DEGs between EVs-IVP and EVs-IVV. The significantly pathways were classified as biological process (BP), cellular component (CC) and molecular function (MF). Non biological process was up regulated by EVs-IVP in bEEcs compared to EVs-IVV.

Table 1S. Number of reads sequence per sample and Mapping rates. More than 93% of the setof reads of each sample aligned one time and less than 4% aligned more than 1 time, with a total rate of alignment more than 98% reflecting the accurate of assembly using the genome reference ARS-VCD1.2

Sample ID	Total Reads	Reads aligned 1 time	Reads aligned > 1 time	Total Aligment Rate
Control1	28474603	27051719 (95.00%)	933916 (3.28%)	99.07%
Control2	23415608	22301271 (95.24%9	768176 (3.28%)	99.18%
Control3	28609208	27179707 (95.00%)	953299 (3.33%)	99.13%
Control4	21993991	20566496 (93.51%)	822725 (3.74%)	98.59%
Control5	25892496	24243743 (93.63%)	891009 (3.44%)	98.47%
EVs- IVV1	27239389	25600687 (93.98%)	950207 (3.49%)	98.68%
EVs- IVV2	24962780	23549901 (94.34%)	822727 (3.30%)	98.77%
EVs- IVV3	26056250	24559132 (94.25%)	859209 (3.30%)	98.73%
EVs- IVP1	24351896	23181076 (95.19%)	800772 (3.29%)	99.16%
